# Risk of Cancer Death Among White, Black, and Hispanic Populations in South Florida

**DOI:** 10.5888/pcd16.180529

**Published:** 2019-06-27

**Authors:** Paulo S. Pinheiro, Karen E. Callahan, Tulay Koru-Sengul, Justine Ransdell, Layla Bouzoubaa, Clyde P. Brown, Erin Kobetz

**Affiliations:** 1 Sylvester Comprehensive Cancer Center, Department of Public Health Sciences, University of Miami School of Medicine, Miami, Florida; 2 School of Community Health Sciences, University of Nevada Las Vegas, Las Vegas, Nevada; 3 Department of Public Health Sciences, University of Miami School of Medicine, Miami, Florida; 4 College of Pharmacy and Pharmaceutical Sciences, Florida A&M University, Tallahassee, Florida

## Abstract

**Background:**

The cancer burden in South Florida, with a population of more than 6 million with a heavily Hispanic and large Afro-Caribbean population, has not been quantified.

**Methods:**

We analyzed 2012–2016 cancer mortality data from South Florida for white, Hispanic, and black populations with disaggregation for Cuban, Puerto Rican, South American, African American, and Afro-Caribbean groups. We calculated cancer site-specific and all-sites combined age-adjusted mortality rates, and we used negative binomial regression to determine mortality rate ratios to compare South Florida’s cancer mortality rates with those of the rest of the nation.

**Results:**

We analyzed 53,837 cancer deaths. Per 100,000 population, cancer mortality rates in South Florida were similar among white (173 per 100,000) and black (176 per 100,000) men and among white and black women (133 for both), and they were lowest among Hispanic men (151 per 100,000) and women (93 per 100,000). However, compared with their counterparts nationally, Hispanic residents in South Florida had higher cancer mortality rates, largely driven by Cuban residents, and mortality rates among white and black residents, especially male residents, were substantially lower. Liver cancer rates were high among white and Puerto Rican “baby boomers”; lung cancer mortality was low among all groups except Cuban men; cervical cancer was high among white, black, and Puerto Rican women.

**Conclusion:**

Cancer patterns are not monochromatic in all US regions; South Florida is distinctive. Meeting the needs of an aging diverse population presents challenges for all major metropolitan areas. Expanding surveillance, increasing minority participation in clinical trials, and investing in culturally specific community-based health promotion must continue.

**Summary**

**What is already known on this topic?**

Compared with white Americans, cancer mortality rates are higher among black Americans and lower among Hispanic Americans.

**What is added by this report?**

White and black residents of South Florida have lower cancer mortality rates than their national counterparts, and the advantage for black South Floridians results from the large number of Afro-Caribbean residents, who have low risk for cancer mortality. Hispanics in South Florida, most of whom are Cuban, have higher mortality rates for most cancers than their national counterparts.

**What are the implications for public health practice?**

Effective cancer prevention and control efforts should be specific to populations to account for diversity.

## Introduction

As cancer prevalence rises among the increasingly diverse US population, targeted and culturally specific community-based cancer prevention and control efforts are required. Ideally, these will be based on the most current knowledge of the population-specific cancer burden, including disparities, for a specific geographic area (1). South Florida is the eighth largest metropolis in the United States and is growing; it has more than 6 million residents and comprises nearly one-third of Florida’s total population. South Florida is a gateway to Latin America and the Caribbean, and demographics reflect this: 44% of residents are Hispanic, 32% are white, and 21% are black (2). The diversity extends intra-racially; 33% of black residents in South Florida are Afro-Caribbean, many from Haiti and Jamaica (2). Likewise, the Hispanic population in South Florida, born primarily outside of the continental United States, differs vastly from the predominantly Mexican-origin Hispanic US population. Cubans are the largest Hispanic group in South Florida, also home to large South American, Central American, and Puerto Rican populations (2).

Cancer is the leading cause of death for Hispanics and Afro-Caribbeans in Florida (3,4) as well as elsewhere in the United States (5,6). Yet no studies to date have analyzed cancer death rates in South Florida, the catchment area of the Sylvester Comprehensive Cancer Center at the University of Miami, which includes Broward, Miami-Dade, Monroe, and Palm Beach counties. Thus, our objective was to characterize patterns of cancer deaths by race/ethnicity in South Florida and compare rates between South Florida and the United States. We hypothesized that the cancer mortality rates of metropolitan areas would differ from those of the United States as a whole.

## Methods

We analyzed 5 years of cancer mortality data (January 1, 2012, through December 31, 2016) for Florida residents in the Miami-Dade-Broward-Palm Beach metropolitan statistical area. Data were obtained from the Florida Department of Health Vital Statistics for this cross-sectional study. Deaths from Monroe County (n = 956) were not included because of lack of corresponding population-specific denominators. Cancer sites were coded according to the *International Classification of Diseases, 10th Revision*. We analyzed common causes of cancer death (lung, breast, prostate, colorectum, pancreas, endometrium, ovary, and liver); cancers with high mortality rates among Hispanic and black populations (cervical, stomach, and multiple myeloma); and all-sites-combined (ie, deaths from all cancers). Because of the high prevalence of premenopausal breast cancer among black women (3,5), breast cancer was analyzed in 2 categories, using an age threshold of 50 to approximate premenopausal and postmenopausal breast cancer (7). Liver cancer is common among minorities (8), and recent reports from California (9) and New York (5) have indicated significant differences between people born before 1945 and the 1945–1965 birth cohort, which is subject to the Centers for Disease Control and Prevention’s (CDC’s) recommendation for one-time hepatitis C (HCV) testing because of high HCV prevalence (10). To approximate these 2 groups, we analyzed liver cancer rates for people aged 50 to 69 years and for those aged 70 years or older.

We carefully examined codes for race/ethnicity and birthplace, including text fields, to accurately classify race/ethnicity (3,5,11). We analyzed the total Hispanic population and separately analyzed the Cuban, Puerto Rican, South American, and Other Hispanic (Mexican, Dominican, Central American, and Spaniard) populations. We analyzed the total non-Hispanic black population (hereafter referred to as black) and separately analyzed the African American (black, born in the United States) and Afro-Caribbean (black, born in the Caribbean) populations. Finally, we analyzed the non-Hispanic white population (hereafter referred to as white). Black decedents born in Africa or other countries not studied here were included in the aggregate black rates but not studied separately (n = 225). Also, because of low numbers, American Indian/Alaska Native, Asian, and Pacific Islander decedents were not included in this study (n = 714).

We obtained population denominators for the tri-county region for each year from 2012 to 2016 from the US Census Bureau’s American Community Survey (2), assembled into populations corresponding with numerator data and aggregated. Hispanics who were “not otherwise specified” from the census data (less than 2%) were bridged to each study population proportionately by age group and sex.

Sex-specific, annualized, age-standardized (2000 US standard population) cancer mortality rates for 2012–2016 were calculated per 100,000 people using 18 age group bands, all 5-year except the last (ie, aged ≥85).

For direct comparison with South Florida estimates, national cancer mortality rates for 2012 through 2014 were obtained from CDC (11) (South Florida data were restricted to these 3 years for comparability). Age-adjusted site-specific mortality rate ratios (MRRs) and corresponding 95% confidence intervals (CIs) were computed for white, black, and Hispanic populations in South Florida, using their corresponding racial/ethnic counterparts nationally as the reference population. To account for the large number of foreign-born black decedents in South Florida, MRRs for the aggregate black as well as the exclusively African American populations were both computed. MMRs were computed using negative binomial regression. Models included decedents aged 35 or older, except for prostate cancer and multiple myeloma, which included decedents aged 45 or older.

Data management and statistical analyses were performed using SPSS version 22.0 (IBM Corporation) and SAS version 9.4 (SAS Institute, Inc). Ethical review resulting in exempt status was obtained from the University of Miami institutional review board.

## Results

In 2012–2016, cancer was the primary cause of death for 53,837 South Florida residents, of whom 29,050 were white (54%), 16,918 Hispanic (31%), and 7,869 black (15%). The median age of white decedents was 48 years; Hispanic decedents, 38 years; and black decedents, 31 years. Ninety-five percent of Hispanic, 41% of black, and 12% of white decedents were born outside the continental US (including Puerto Rico) (Table 1).

**Table 1:** Population Characteristics, Study on Patterns of Cancer Deaths by Race/Ethnicity in South Florida, 2012–2016

Characteristic	Population Data (2)			Cancer Data		
	Total Population	Median Household Income of Population, $	Median Age of Population, y	No. of Cancer Deaths	Foreign-Born Decedents, %^a^	Top Country of Birth of Decedents, %
White	1,889,467	79,400	48	29,050	12	United States, 88
Black, all^b^	1,237,215	46,700	31	7,869	41	United States, 59
African American	811,825	45,000	24	4,633	0	United States, 100
Afro-Caribbean	403,273	48,000	42	3,011	100	Haiti, 48
Hispanic, all	2,601,132	55,500	38	16,918	95	Cuba, 63
Cuban	1,107,542	54,100	43	10,920	98	Cuba, 98
South American	556,275	60,000	38	2,670	99	Colombia, 38
Puerto Rican	241,947	59,000	33	1,220	79	Puerto Rico, 79
Other Hispanic^c^	695,368	51,225	33	2,108	91	Nicaragua, 23

a Born outside of the 50 US states; includes birth on Island of Puerto Rico.

b Includes people of black/African descent not born in Caribbean countries or the United States.

c Includes decedents from Mexico, Central America, the Dominican Republic, and Spain.

Lung cancer was the leading cause of cancer death for all male groups except Afro-Caribbeans and other Hispanics, for whom prostate cancer was the leading cause followed by lung and colorectal cancers. Prostate and colorectal cancers were the second and third leading causes of cancer death for all other male groups, apart from Puerto Ricans for whom liver cancer was the second leading cause of cancer death, followed by prostate. Among female decedents, lung and breast cancers were the first or the second leading cause of cancer death in all groups, followed by colorectal cancer as the third, except for Afro-Caribbeans, for whom both breast and colorectal cancers preceded lung cancer (Table 2).

**Table 2:** Age-Adjusted^a^ Mortality Rates for Selected Cancer Sites, Study on Patterns of Cancer Deaths by Race/Ethnicity in South Florida, 2012–2016

Cancer type	White	Black			Hispanic				
		All Black	African American	Afro-Caribbean	All Hispanic	Puerto Rican	Cuban	South American	Other Hispanic^b^
	Age-Adjusted Mortality Rate Per 100,000 (95% Confidence Interval)								
**Male**									
Stomach	2.9 (2.5–3.3)	7.8 (6.5–9.4)	7.8 (5.9–10.0)	7.8 (5.9–10.4)	4.9 (4.3–5.6)	6.7 (4.2–10.2)	3.9 (3.2–4.7)	7.4 (5.6–9.7)	5.2 (3.6–7.2)
Colorectal	15.4 (14.5–16.5)	19.6 (17.5–21.9)	22.6 (19.5–26.1)	15.4 (12.7–18.6)	15.0 (13.9–16.1)	12.4 (9.0–16.6)	16.0 (14.6–17.5)	13.1 (10.5–15.9)	13.0 (10.2–16.4)
Liver^c^	8.8 (8.1–9.5)	9.6 (8.2–11.2)	11.2 (9.1–13.7)	7.0 (5.2–9.4)	7.6 (6.9–8.4)	14.9 (11.1–19.7)	6.4 (5.5–7.4)	8.3 (6.2–10.8)	10.3 (7.8–13.4)
Aged 50–69 y	4.5 (4.0–5.0)	4.0 (3.3–4.9)	5.7 (4.5–7.2)	1.9 (1.2–3.3)	3.0 (2.5–3.4)	6.2 (4.2–8.9)	2.6 (2.1–3.3)	1.4 (0.8–2.3)	4.5 (3.2–6.0)
Aged ≥70 y	4.0 (3.6–4.5)	4.4 (3.3–5.7)	4.5 (2.9–6.4)	3.7 (2.3–5.8)	4.4 (3.8–5.0)	8.5 (5.4–12.6)	3.6 (2.9–4.3)	6.5 (4.5–8.9)	5.7 (3.5–8.4)
Pancreas	13.7 (12.8–14.6)	10.1 (8.6–11.8)	13.0 (10.6–15.7)	6.9 (5.2–9.3)	11.0 (10.0–11.9)	10.4 (7.2–14.5)	11.5 (10.3–12.8)	11.1 (8.8–13.9)	8.7 (6.3–11.5)
Lung	42.4 (40.9–44.0)	33.9 (31.1–36.9)	47.8 (43.1–52.8)	18.1 (15.3–21.6)	36.3 (34.6–38.0)	31.9 (26.1–38.7)	45.0 (42.7–47.5)	20.6 (17.3–24.2)	19.4 (15.6–23.7)
Prostate	14.6 (13.8–15.5)	37.9 (34.6–41.3)	39.9 (35.2–45.0)	34.4 (30.0–39.3)	17.7 (16.4–18.9)	13.7 (9.6–18.7)	18.1 (16.7–19.7)	15.9 (12.8–19.5)	23.6 (19.0–28.8)
Multiple myeloma	3.0 (2.6–3.4)	6.7 (5.4–8.1)	7.8 (5.9–10.1)	5.4 (3.9–7.6)	3.5 (3.0–4.1)	5.1 (2.9–8.2)	2.9 (2.3–3.6)	3.9 (2.6–5.7)	5.8 (3.8–8.2)
All-sites-combined^d^	172.8 (169.7–176.1)	175.5 (168.8–182.2)	206.3 (196.1–216.7)	137.6 (128.8–147.0)	150.6 (147.2–154.2)	151.4 (138.0–165.6)	163.5 (158.9–168.2)	131.6 (123.0–140.6)	130.4 (120.4–140.8)
**Female**									
Stomach	1.5 (1.2–1.8)	4.6 (3.8–5.5)	4.9 (3.6–6.3)	4.4 (3.3–6.3)	2.8 (2.4–3.2)	2.5 (1.3–4.3)	2.1 (1.6–2.7)	4.5 (3.3–5.9)	4.0 (2.8–5.4)
Colorectal	10.5 (9.8–11.4)	13.9 (12.5–15.5)	17.5 (15.1–20.1)	10.9 (9.2–13.3)	10.1 (9.4–10.9)	10.1 (7.4–13.4)	12.0 (10.9–13.2)	7.1 (5.6–8.9)	7.8 (6.1–9.8)
Liver^c^	3.8 (3.3–4.3)	4.3 (3.5–5.2)	5.2 (4.0–6.6)	3.3 (2.4–5.1)	4.2 (3.7–4.7)	4.7 (2.8–7.3)	3.6 (3.0–4.3)	5.1 (3.9–6.7)	6.4 (4.9–8.2)
Aged 50–69 y	1.5 (1.2–1.8)	1.7 (1.3–2.2)	2.6 (1.8–3.5)	0.9 (0.5–2.4)	1.1 (0.8–1.3)	0.7 (0.2–1.8)	0.9 (0.6–1.4)	1.0 (0.6–1.7)	1.6 (1.0–2.5)
Aged ≥70 y	2.1 (1.8–2.5)	2.3 (1.7–3.0)	2.3 (1.5–3.4)	2.0 (1.3–3.7)	2.9 (2.5–3.3)	4.0 (2.3–6.5)	2.3 (1.9–2.8)	3.9 (2.7–5.3)	4.5 (3.2–6.1)
Pancreas	10.0 (9.3–10.8)	10.1 (8.9–11.5)	12.7 (10.6–15.0)	7.3 (5.9–9.5)	7.1 (6.5–7.8)	8.2 (5.8–11.3)	7.2 (6.4–8.1)	7.2 (5.8–9.0)	7.0 (5.4–8.9)
Lung	34.8 (33.4–36.1)	18.5 (16.8–20.3)	28.8 (25.7–32.1)	9.1 (7.5–11.3)	14.4 (13.5–15.3)	14.1 (10.8–18.1)	16.5 (15.3–17.9)	14.0 (12.0–16.4)	9.7 (7.8–11.8)
Breast	20.2 (19.0–21.4)	25.7 (23.7–27.7)	30.7 (27.6–34.0)	20.5 (18.1–23.5)	14.6 (13.7–15.5)	16.2 (12.7–20.3)	15.5 (14.2–16.9)	12.4 (10.6–14.5)	13.1 (11.0–15.4)
Premenopausal^e^	3.5 (2.9–4.3)	5.3 (4.4–6.4)	5.4 (4.1–7.0)	5.2 (4.0–7.2)	2.3 (1.9–2.7)	1.9 (0.8–3.5)	2.2 (1.6–3.2)	2.3 (1.6–3.2)	2.6 (1.8–3.6)
Postmenopausal	16.6 (15.7–17.6)	20.3 (18.6–22.1)	25.3 (22.4–28.3)	15.3 (13.3–17.9)	12.3 (11.5–13.2)	14.3 (11.0–18.3)	13.3 (12.2–14.5)	10.1 (8.5–12.1)	10.5 (8.7–12.7)
Cervix	2.8 (2.3–3.3)	4.0 (3.2–4.8)	4.9 (3.7–6.4)	3.0 (2.1–4.6)	1.9 (1.6–2.3)	2.9 (1.6–4.8)	1.8 (1.4–2.5)	1.9 (1.2–2.8)	1.9 (1.2–2.9)
Endometrium	4.3 (3.8–4.8)	9.2 (8.1–10.5)	9.8 (8.0–11.7)	8.6 (7.1–10.7)	3.8 (3.3–4.3)	4.3 (2.6–6.6)	4.0 (3.3–4.7)	3.1 (2.2–4.2)	4.3 (3.1–5.8)
Ovary	7.3 (6.6–8.0)	5.7 (4.8–6.7)	6.0 (4.7–7.6)	5.3 (4.1–7.1)	4.9 (4.4–5.5)	5.5 (3.5–8.3)	5.6 (4.8–6.4)	4.8 (3.7–6.2)	3.3 (2.3–4.6)
Multiple myeloma	2.0 (1.7–2.3)	4.2 (3.4–5.1)	3.7 (2.6–5.0)	4.6 (3.5–6.5)	2.0 (1.6–2.3)	2.3 (1.2–4.2)	1.9 (1.5–2.4)	2.4 (1.6–3.5)	1.6 (1.0–2.6)
All-sites-combined^d^	132.6 (129.9–135.4)	133.4 (128.9–138.1)	164.1 (156.7–171.8)	106.2 (99.5–113.4)	92.9 (90.6–95.2)	103.0 (93.8–112.9)	97.1 (93.9–100.4)	91.6 (86.2–97.2)	84.5 (78.8–90.4)

a Adjusted to the US 2000 standard population.

b Includes decedents from Mexico, Central America, the Dominican Republic, and Spain.

c Includes all ages.

d All-sites-combined includes all cancers, including those not listed here.

e Menopausal status approximated by use of age threshold of 50.

African American male and female decedents had the highest all-sites-combined cancer mortality rate among all groups, as well as the highest rates for most cancers. Compared with white male and female decedents in South Florida, all 4 disaggregated Hispanic groups as well as Afro-Caribbeans had lower overall rates. Hispanics in aggregate had significantly higher mortality rates than white South Floridians only for stomach (male and female) and prostate cancers (*P* < .05). Afro-Caribbeans had significantly higher rates compared with whites for stomach, prostate, multiple myeloma, premenopausal breast, and endometrial cancers, but lower rates for most other cancers, particularly lung cancer (Table 2).

For Hispanics, considerable heterogeneity was found in cancer mortality rates. Liver cancer among Puerto Rican male decedents was 15.0 per 100,000, compared with the lower rate of 6.4 among Cubans and a rate of 8.8 per 100,000 among white decedents (reference group) in South Florida. Conversely, lung cancer rates for Cuban male decedents were higher than for white male decedents, 45.0 compared with 42.4 per 100,000, whereas all female Hispanic groups and male South American decedents and other Hispanic decedents had markedly lower lung cancer rates than whites. Cuban decedents also had high colorectal cancer rates. Low mortality rates for most cancers were observed in South Americans and the heterogeneous other Hispanics, except for stomach, prostate, and liver (especially aged ≥70 y) cancers (Table 2).

In comparison with the overall US Hispanic population, the all-sites-combined risk of cancer death in South Florida was 11% higher among Hispanic males (MRR, 1.11; 95% CI, 1.08–1.14) and approximately equivalent among Hispanic females. Conversely, compared with their corresponding counterparts nationally, risk of cancer death in South Florida was significantly lower among white males (10% lower), white females (5% lower), black males (27% lower), and black females (19% lower) (Table 3). Comparison of South Florida’s African American population to the overall US black population reduced the risk advantage from 27% to only 8% lower mortality for males, and no significant difference was found between African American females in South Florida and black females in the United States (Table 3).

**Table 3:** Mortality Rate Ratios^a^ for Selected Cancer Sites, by Race/Ethnicity, South Florida Compared With US Counterparts, Study on Patterns of Cancer Deaths by Race/Ethnicity in South Florida, 2012–2014

Cancer type	White (Ref: US white)	All Black (Ref: US black)	African American (Ref: US black)	All Hispanic (Ref: US Hispanic)
	Mortality Rate Ratio (95% Confidence Interval)			
**Male**				
Stomach	0.84 (0.71–0.99)	0.94 (0.76–1.15)	1.00 (0.74–1.32)	0.79 (0.68–0.92)
Colorectal	0.90 (0.83–0.98)	0.83 (0.73–0.94)	1.08 (0.93–1.27)	1.06 (0.97–1.16)
Liver, aged 50–69 y	1.23 (1.09–1.40)	0.48 (0.38–0.61)	0.69 (0.52–0.91)	0.48 (0.40–0.58)
Liver, aged ≥70 y	1.04 (0.91–1.19)	0.75 (0.53–1.06)	0.81 (0.50–1.31)	0.64 (0.53–0.76)
Pancreas	1.05 (0.91–1.13)	0.64 (0.54–0.77)	0.86 (0.69–1.07)	1.15 (1.03–1.28)
Lung	0.80 (0.77–0.83)	0.56 (0.51–0.61)	0.80 (0.72–0.89)	1.46 (1.38–1.55)
Prostate	0.81 (0.75–0.80)	0.99 (0.89–1.09)	1.16 (1.01–1.32)	1.16 (1.06–1.26)
Multiple myeloma	0.79 (0.68–0.92)	0.98 (0.79–1.21)	1.00 (0.74–1.36)	1.09 (0.90–1.32)
All-sites-combined^b^	0.90 (0.87–0.91)	0.73 (0.70–0.76)	0.92 (0.87–0.97)	1.11 (1.08–1.14)
**Female**				
Stomach	0.94 (0.77–1.14)	1.10 (0.87–1.39)	1.19 (0.87–1.66)	0.79 (0.67–0.94)
Colorectal	0.88 (0.82–0.95)	0.89 (0.78–1.01)	1.00 (0.84–1.19)	1.15 (1.05–1.26)
Liver, aged 50–69 y	1.28 (1.02–1.60)	0.68 (0.48–0.98)	1.10 (0.73–1.65)	0.56 (0.42–0.74)
Liver, aged ≥70 y	1.06 (0.90–1.24)	0.83 (0.57–1.19)	0.71 (0.40–1.26)	0.70 (0.59–0.84)
Pancreas	1.04 (0.97–1.13)	0.73 (0.62–0.85)	0.90 (0.73–1.11)	0.98 (0.88–1.09)
Lung	0.96 (0.89–1.03)	0.58 (0.52–0.64)	0.91 (0.80–1.02)	1.13 (1.04–1.22)
Breast	0.94 (0.88–0.99)	0.89 (0.81–0.98)	1.12 (0.99–1.26)	1.06 (0.98–1.14)
Cervix	1.24 (1.02–1.50)	1.34 (1.08–1.66)	1.71 (1.30–2.23)	0.92 (0.75–1.13)
Endometrium	1.00 (0.88–1.13)	1.04 (0.89–1.23)	1.12 (0.90–1.40)	1.05 (0.90–1.22)
Ovary	0.99 (0.90–1.09)	0.89 (0.73–1.09)	1.02 (0.78–1.33)	1.02 (0.90–1.16)
Multiple myeloma	0.94 (0.81–1.11)	0.77 (0.61–0.97)	0.73 (0.52–1.03)	0.97 (0.79–1.18)
All-sites-combined^b^	0.95 (0.91–0.98)	0.81 (0.78–0.85)	1.01 (0.95–1.06)	0.99 (0.96–1.03)

Abbreviations: CI, confidence interval; MMR, mortality rate ratio; Ref, reference group.

a MMRs derived from negative binomial regression including ages ≥35 except prostate and multiple myeloma, ≥45, and liver as described.

b All-sites-combined includes all cancers, including those not listed here.

Compared with national data, risk of death from lung cancer was 20% lower for white and African American males in South Florida but 46% and 13% higher for Hispanic males and females, respectively. Compared with their US counterparts, the cervical cancer mortality rate in South Florida was 24% higher among whites and 71% higher among African Americans (Table 3). Furthermore, white males had a 19% lower prostate cancer rate and a 10% lower colorectal cancer rate, and white females had a 12% lower colorectal cancer rate and a 6% lower breast cancer rate than their national counterparts.

The risk of liver cancer mortality among white males and females in the age 50–69 group was significantly higher than their US counterparts (23% and 28% higher in males and females, respectively). For Hispanics aged 50 to 69 years, compared with their US counterparts, substantial risk of death for lung cancer, prostate cancer (16% higher), pancreatic cancer in males (15% higher), and colorectal cancer in females (15% higher) was offset by a reduced risk of liver cancer mortality, 52% and 44% lower for males and females, respectively. Even among decedents aged 70 years or older, liver cancer mortality was 36% lower for Hispanic men and 30% lower for Hispanic women than it was nationally (Table 3).

## Discussion

Although South Florida cancer patterns generally align with the epidemiology of cancer in the United States (ie, highest rates for African Americans, lowest rates for Hispanics, and rates for whites in between [5,12,13]), metropolitan-area–specific cancer mortality rate disparities are evident. The densely populated and increasingly diverse South Florida region has several unique aspects to its cancer burden.

Contrary to typical findings in the United States, no disparity in overall cancer mortality was found between South Florida’s aggregate black and white populations. Moreover, compared with their counterparts nationally, white and black populations in South Florida had lower cancer mortality rates and Hispanics had significantly higher rates. Careful scrutiny of the unique racial/ethnic composition of South Florida’s black and Hispanic populations, combined with a portrayal of the specific characteristics of the white population, can provide perspective on these findings.

In this study, the low rates of cancer mortality among South Florida’s black population are almost entirely driven by the inclusion of low-risk Afro-Caribbeans, who make up 33% of South Florida’s black (and 7% of the total) population (2). Analyzed separately, African Americans in South Florida experience cancer mortality rates similar to those of African Americans in the United States, with one notable exception: the lung cancer mortality rate was 20% lower than among their black counterparts nationally, an unexpected but encouraging finding. Although it is possible that African American men in South Florida have a low prevalence of current and/or past smoking, this information is not currently available by racial/ethnic group at a local level. Research is warranted, as lung and other tobacco-driven cancers account for a sizable proportion of all cancer deaths. Among Afro-Caribbeans in South Florida, the lower cancer mortality is consistent with prior studies from Florida (3,4) and New York (5). Likewise, the previously reported (3,5) higher race-specific rates among both Afro-Caribbean and African American populations for endometrial, prostate, premenopausal breast, and multiple myeloma cancers were confirmed for South Florida’s black population in this study.

South Florida’s 33% white population had lower rates than their counterparts nationally for all 4 leading causes of cancer death: lung, prostate, breast, and colorectal. Socioeconomic indicators likely explain this finding. The older white population had a median household income of $79,400 and a 67% prevalence of college education during the study period, higher than the national $72,000 and 54%, respectively. The favorable impact of higher socioeconomic status on cancer indicators has been well documented (14–16). Higher income and education level in the United States have generally been linked to lower prevalence of cancer risk factors, including obesity (17) and smoking (18), resulting in lower cancer incidence and better access to quality health care (19), including cancer screening and high-quality cancer treatment. In South Florida, lower incidence and higher survival combined likely explain the lower mortality rates among whites. Nonetheless, the overall cancer burden is high, with nearly 6,000 deaths annually, because of the large number of older whites in the region. Moreover, the markedly higher liver cancer mortality among the white “baby boomers” (ie, roughly equivalent to people aged 50–69 years, approximating the cohort with high HCV prevalence) in South Florida merits special attention. Priority should be placed on increasing compliance with one-time screening for people born between 1945 and 1965 as recommended by CDC, given that early identification and treatment of HCV infection can prevent liver cancer (10).

The largest racial/ethnic group in South Florida is Hispanic, comprising 45% of the total population. The Hispanic cancer mortality rate in this region is predominantly driven by the Cuban population, who are not only older (median age among Hispanics, 43 y) but also have higher overall cancer mortality than other Hispanic groups (4). Cubans are appreciably distinct from other Hispanic groups, with a comparatively higher burden of colorectal, lung, prostate, and breast cancers but markedly lower rates of cancers caused by infectious agents (ie, cervical, liver, and stomach). Lung cancer rates among Cuban males in South Florida surpass even those of whites, which may reflect the previously documented high prevalence of smoking among Cuban males (20). Conversely, liver cancer mortality is low for Cubans, with patterns distinct from other racial/ethnic groups in South Florida. Cubans appear to have neither the increased susceptibility for HCV-related liver cancer documented for many Americans in the 50–69 age range, nor the high rates typically seen among immigrant Hispanics older than 70. In contrast, Puerto Rican men in South Florida have a high liver cancer mortality rate; this is true both for those aged 50 to 69 years, concordant with the high prevalence of HCV among Puerto Ricans (21,22), and for those older than 69 years, consistent with the liver cancer mortality profile for Puerto Ricans in New York (5). Notably, neither Hispanics in general, nor Puerto Ricans in particular, are prioritized as a target group for intervention in the current National Viral Hepatitis Plan (9); this oversight should be re-examined.

The high prostate cancer mortality rate among Hispanics compared with whites in South Florida and among African Americans in South Florida in relation to US blacks is puzzling. Research is needed to ascertain whether this stems from a truly higher risk of aggressive prostate cancer among these groups or from survival disparities resulting from differential access to quality health care for prostate cancer, which requires a complex treatment protocol (23).

Our population-based study benefits from its accurate disaggregation of Hispanic and black decedents into distinct groups with unique characteristics that affect their cancer burden, accomplished by having more than 99% of exact birthplace information and specific race/ethnicity codes. Nonetheless, the limitations customarily found in descriptive epidemiological studies apply. Neither risk factor data nor screening and treatment information at the individual level are available from mortality data. We examined cancer mortality, a function of cancer incidence and survival. Although our results are consistent with previously reported cancer incidence patterns for Hispanic groups (24), limited health care access and poor health care quality for some Hispanics may have resulted in poor cancer survival, thus affecting the mortality burden. Unfortunately, the accuracy of survival data among the foreign-born is prone to biases arising from passive follow-up methods in cancer registries and missing information on birthplace or Hispanic group (25,26). Future studies should attempt to improve survival estimation methods for foreign-born, mostly minority populations.

This study shows heterogeneity in the cancer profile of South Florida, indicated by examining differences by distinct racial/ethnic groups: the divergent liver cancer burden between Cuban and Puerto Rican males is one such example. Addressing the cancer prevention and control needs of this diverse population requires leveraging this information in all community outreach and engagement efforts, which could include targeted smoking interventions for Cuban men, HCV testing and antiviral treatment especially for Puerto Rican men and white “baby boomers,” and a continued focus on cervical cancer screening among all South Florida women. Moreover, researchers have a unique opportunity in South Florida to study the biologic and genetic mechanisms driving race-specific vulnerability for certain cancers among South Florida’s black population, given its 2 distinct birthplace cohorts, African Americans and Afro-Caribbeans.

Systematic collection of population-specific data taking into account the area’s extreme heterogeneity, as occurs in other equally diverse areas of the country such as California (27) and New York City (28), is lacking in Florida. Although some group-specific cancer research has been conducted in the area, these studies relied primarily on relatively small samples (29,30). Cubans are most disadvantaged by this gap in surveillance, as general data collected on US Hispanics is insufficient for meeting their needs. The young composition of South Florida’s burgeoning population, which comprises primarily Hispanics and blacks, provides opportunities for community-based and culturally specific lifestyle interventions, especially among socioeconomically disadvantaged groups, including Central Americans and Haitians, to control South Florida’s future cancer burden. The unique cancer control challenges facing South Florida, like other large US metropolitan areas, require expansion of population-specific surveillance, increased minority participation in clinical trials, and continued investment in community health education and promotion.
